# The Role of Gut Microbiota in Duodenal-Jejunal Bypass Surgery-Induced Improvement of Hepatic Steatosis in HFD-Fed Rats

**DOI:** 10.3389/fcimb.2021.640448

**Published:** 2021-04-02

**Authors:** Yi Gao, Jia Zhang, Xiao Xiao, Yifan Ren, Xiaopeng Yan, Jing Yue, Tieyan Wang, Zheng Wu, Yi Lv, Rongqian Wu

**Affiliations:** ^1^ National Local Joint Engineering Research Center for Precision Surgery and Regenerative Medicine, Shaanxi Provincial Center for Regenerative Medicine and Surgical Engineering, First Affiliated Hospital of Xi’an Jiaotong University, Xi’an, China; ^2^ Department of Hepatobiliary Surgery, First Affiliated Hospital of Xi’an Jiaotong University, Xi’an, China; ^3^ Gastrointestinal Surgery Department, Affiliated Hospital of Guilin Medical University, Guilin, China; ^4^ School of Basic Medicine, Hubei University of Medicine, Shiyan, China; ^5^ Department of Pathology, Shiyan Taihe Hospital, Hubei University of Medicine, Shiyan, China

**Keywords:** gut-liver axis, gut microbiota, NAFLD, duodenal-jejunal bypass, fecal microbiota transplantation

## Abstract

Bariatric surgery including duodenal-jejunal bypass surgery (DJB) improves insulin sensitivity and reduces obesity-associated inflammation. However, the underlying mechanism for such an improvement is still incompletely understood. Our objective was to investigate the role of the gut microbiota in DJB-associated improvement of hepatic steatosis in high fat diet (HFD)-fed rats. To study this, hepatic steatosis was induced in male adult Sprague-Dawley rats by feeding them with a 60% HFD. At 8 weeks after HFD feeding, the rats were subjected to either DJB or sham operation. HFD was resumed 1 week after the surgery for 3 more weeks. In additional groups of animals, feces were collected from HFD-DJB rats at 2 weeks after DJB. These feces were then transplanted to HFD-fed rats without DJB at 8 weeks after HFD feeding. Hepatic steatosis and fecal microbiota were analyzed at 4 weeks after surgery or fecal transplantation. Our results showed that DJB alleviated hepatic steatosis in HFD-fed rats. Fecal microbiota analysis showed that HFD-fed and standard diet-fed rats clustered differently. DJB induced substantial compositional changes in the gut microbiota. The fecal microbiota of HFD-fed rats received fecal transplant from DJB rats overlapped with that of HFD-DJB rats. Treatment of rats with HFD-induced liver lesions by fecal transplant from DJB-operated HFD-fed rats also attenuated hepatic steatosis. Thus, alterations in the gut microbiota after DJB surgery are sufficient to attenuate hepatic steatosis in HFD-fed rats. Targeting the gut microbiota could be a promising approach for preventing or treating human NAFLD.

## Introduction

Nonalcoholic fatty liver disease (NAFLD) is the most common liver disorder worldwide and a major risk factor for the development of end-stage liver disease and hepatocellular carcinoma (HCC) (Sanyal, 2019; Benhammou et al., 2020; Lazarus et al., 2020). It ranges in severity from simple steatosis (excessive fat accumulation) to nonalcoholic steatohepatitis (NASH, liver cell injury and inflammation). NASH may progress to liver fibrosis, cirrhosis, and eventually decompensated liver disease. As increased insulin resistance underlies most cases of NAFLD, it is considered to be the hepatic manifestation of metabolic syndrome and is strongly associated with obesity, type 2 diabetes, cardiovascular disease and chronic renal impairment (Rinella, 2015; Mahady and Adams, 2018). With the rising prevalence of diabetes and obesity worldwide, NAFLD is predicted to become the most frequent indication for liver transplantation in the next decade (Sayiner and Younossi, 2019).

Bariatric surgery induces weight loss and restores metabolic homeostasis in obese patients by making changes to their digestive system. Duodenal-jejunal bypass surgery (DJB), a modified Roux-en-Y gastric bypass surgery (RYGB), preserves the stomach volume and bypasses the entire duodenum and the proximal jejunum. An increasing number of clinical and experimental studies have demonstrated that DJB results in significant weight loss and durable glycemic control in rodents and humans with type 2 diabetes (Rubino and Marescaux, 2004; Cohen et al., 2012; Klein et al., 2012; Jurowich et al., 2013; Petry et al., 2015; Kavalkova et al., 2016; Li et al., 2016; Yu et al., 2019; Angelini et al., 2020; Ueno et al., 2020). Recently, several studies have revealed that bariatric surgery including DJB can improve hepatic insulin sensitivity and reduce obesity-associated inflammation (Han et al., 2014; Bower et al., 2015; Lassailly et al., 2015; Shang et al., 2016; Angelini et al., 2020). However, the underlying basis for such an improvement is still incompletely understood.

The gastrointestinal tract harbors a large number of bacteria. It is now well known that this “microbial organ” is known to modulate a variety of pathophysiological functions, including the regulation of energy storage, lipid and choline metabolism, endogenous ethanol production, immune balance, and inflammation (Vijay-Kumar et al., 2010; Mehal, 2013; Rowland et al., 2018; Chen et al., 2020; Jensen et al., 2021; Menni et al., 2021; Stacy et al., 2021). A recent study has shown that a gut microbiota-driven activation of intrahepatic B cells leads to hepatic inflammation and fibrosis during the progression of NASH (Barrow et al., 2021). Altered gut microbiota leads to changes in overall bile acid levels and specific bile acid metabolites, which contribute to enterohepatic tumorigenesis (Sun et al., 2021). Based on clinical as well as animal studies, it is becoming increasingly evident that the gut microbiota is closely related to the development and progression of NAFLD (Schnabl and Brenner, 2014; Quigley and Monsour, 2015; Leung et al., 2016; Mardinoglu et al., 2018; Wei et al., 2018; Yuan et al., 2019; Aron-Wisnewsky et al., 2020). We therefore speculated that compositional changes in the gut microbiota after DJB contribute to the attenuation of hepatic steatosis in NAFLD. To test this hypothesis, we first studied the effect of DJB on hepatic steatosis in high fat diet (HFD)-fed rats, then analyzed changes in the gut microbiota after DJB, finally investigated the effect of a fecal transplant from DJB rats to HFD-fed rats on hepatic steatosis. The main purpose was to explore the role of the gut microbiota in DJB-associated improvement of hepatic steatosis in HFD-fed rats.

## Materials And Methods

### Experimental Animals and Diet

Male Sprague-Dawley rats (200-230g) were obtained from the Experimental Animal Center of Xi’an Jiaotong University. All rats were kept under standard conditions of humidity and temperature. After acclimatization for one week, the rats were fed with either a standard rat chow diet (control diet, CD, SPF-03 grade; Protein:28% Kcal, Fat: 12% Kcal, Carbohydrate: 60% Kcal, Energy density: 3.01 Kcal/g; Keaoxieli Food Company, Beijing, China) or a 60% high-fat diet (HFD, D12492, Protein:20% Kcal, Fat: 60% Kcal, Carbohydrate: 20% Kcal, Energy density: 5.21 Kcal/g; Research Diets Inc., New Brunswick, NJ). All animal experiments were performed in accordance with the guidelines of the China Council on Animal Care and Use and approved by the Institutional Animal Care and Use Committee of the Ethics Committee of Xi’an Jiaotong University Health Science Center (2017-609).

### Duodenal-Jejunal Bypass Surgery (DJB)

After HFD feeding for 8 weeks, the rats were subjected to either DJB or sham operation. The DJB procedure was performed as previously described (Rubino and Marescaux, 2004) and illustrated in **Supplementary Figure 1**. Sham DJB operation involved the same procedure as DJB except that transections and re-anastomosis of the gastrointestinal tract were performed at the same sites. Throughout the operation and the first 24 hours after the operation, the animals were kept on an electric blanket to prevent hypothermia. Tap water containing 5% glucose was free access after recovering from anesthesia and enteral nutrition using the total parenteral nutrition (TPN) solution (Enteral Nutritional Suspension; NUTRICIA, Wuxi, China) was provided for 7 days postoperatively. The rats were resumed to HFD at 1 week after DJB or sham operation. The rats were euthanized at 4 weeks after DJB or sham operation. Blood, tissue and colon content samples were harvested.

### Fecal Microbiota Transplantation (FMT)

To investigate the role of the gut microbiota in DJB-associated improvement of hepatic steatosis in HFD-fed rats, feces were collected from HFD-DJB rats at 2 weeks after surgery. These feces were then transplanted to HFD-fed rats without DJB at 8 weeks after HFD feeding (HFD-DJB-Tr). The control rats received transplantation of their own feces (HFD-Auto-Tr). The FMT procedure was performed as previously described (Garcia-Lezana et al., 2018). Briefly, rats were administered omeprazole (50 mg/Kg/d) for 3 days prior to intestinal decontamination in order to facilitate re-colonization with transplanted stool. To achieve intestinal emptying, rats were maintained isolated in fast grills with free access to water, and two oral doses of PEG-4000 of 1mL and 2mL were administered 24h and 12h, respectively, before the transplant (accompanied by 2mL of water each). Recolonization was performed by a single oral gavage with 100 mg of the corresponding fecal pool diluted in 2mL of sterile PBS. After fecal transplantation, the animals were maintained on the HFD for 4 more weeks. Then the blood, tissue and colon content samples were harvested. A previous study has shown that rats received a single fecal transplant was able to capture the transplanted microbiota lasting for at least 3 months (Manichanh et al., 2010).

### Histological Evaluates

Hematoxylin-eosin staining was used to assess liver histology and Oil Red O staining to analyze lipid accumulation. Samples were evaluated by an expert pathologist who was blinded to the intervention condition using morphometrical quantification of steatosis (Kleiner et al., 2005).

### Electron Microscopy

Ultra-thin sections (70 nm) of liver samples were stained with uranyl acetate and lead citrate and examined under a transmission electron microscope (HT7700, Japan). Hepatic ultrastructure evaluations were performed by a single electron microscopist.

### Measurement of Hepatic Triglycerides

Hepatic triglycerides (TG) was measured by a Chemray-240 Automated Chemistry Analyzer (Shenzhen, China) according to the manufacturer’s instruction. The final concentrations of hepatic TG were expressed in mg of TG per g of liver.

### Quantitative Reverse Transcriptase-Polymerase Chain Reaction (qRT-PCR)

Total RNA was extracted from liver tissue samples with the RNAiso Plus Reagent Kit (Takara Bio Inc., Kusatsu, Shiga, Japan). cDNA was generated with the PrimeScript RT Reagent Kit (Takara Bio Inc., Kusatsu, Shiga, Japan) and was amplified with the SYBR Premix Ex TaqTM Kit (Takara Bio Inc., Kusatsu, Shiga, Japan) and the ABI StepOne plus Real-Time PCR System (Applied Biosystems). GAPDH was used as an internal control for normalization, and the relative expression level of the analyzed gene was calculated by the ΔΔCt method. The following primer sets (Takara Bio Inc., Kusatsu, Shiga, Japan) were used: ACC1 (forward, 5’ CAA TCC TCG GCA CAT GGA GA 3’; reverse, 5’ GCT CAG CCA AGC GGA TGT AGA 3’), SCD1 (forward, 5’ ACA TGT CTG ACC TGA AAG CTG AGA A 3’; reverse, 5’ ACG AAC AGG CTG TGC AGG AA 3’), TGF-β (forward, 5’ CAT TGC TGT CCC GTG CAG A 3’; reverse, 5’ AGG TAA CGC CAG GAA TTG TTG CTA 3’), MCP-1 (forward, 5’ CTA TGC AGG TCT CTG TCA CGC TTC 3’; reverse, 5’ CAG CCG ACT CAT TGG GAT CA 3’), ICAM1 (forward, 5’ GCT TCT GCC ACC ATC ACT GTG TA 3’; reverse, 5’ ATG AGG TTC TTG CCC ACC TG 3’), TNF-α (forward, 5’ TTC CAA TGG GCT TTC GGA AC 3’; reverse, 5’ AGA CAT CTT CAG CAG CCT TGT GAG 3’), IL-10 (forward, 5’ CAG ACC CAC ATG CTC CGA GA 3’; reverse, 5’ CAA GGC TTG GCA ACC CAA GTA 3’) and GAPDH (endogenous control; forward 5’ GGC ACA GTC AAG GCT GAG AAT G 3’; reverse, 5’ ATG GTG GTG AAG ACG CCA GTA 3’).

### 16S rRNA Gene Sequencing Procedure

Microbial DNA was extracted from colon content samples using the E.Z.N.A.^®^ Soil DNA Kit (Omega Bio-tek, Norcross, GA, U.S.) according to the manufacturer’s protocol. The V4-V5 region of the bacteria 16S ribosomal RNA gene were amplified by PCR using primers 515F 5’-barcode-GTG CCA GCM GCC GCG G-3’ and 907R 5’-CCG TCA ATT CMT TTR AGT TT-3’, where the barcode is an eight-base sequence unique to each sample. Amplicons were extracted from 2% agarose gels and purified using the AxyPrep DNA Gel Extraction Kit (Axygen Biosciences, Union City, CA, U.S.) according to the manufacturer’s instructions and quantified using QuantiFluor™-ST (Promega, U.S.). Purified PCR products were quantified by Qubit^®^3.0 (Life Invitrogen) and every twenty-four amplicons whose barcodes were different were mixed equally. The pooled DNA product was used to construct Illumina Pair-End library following Illumina’s genomic DNA library preparation procedure. Then the amplicon library was paired-end sequenced (2×250) on an Illumina MiSeq platform (Shanghai BIOZERON Co., Ltd) according to the standard protocols. Raw fastq files were demultiplexed, quality-filtered using QIIME (version 1.17) with the following criteria: (i) The 250 bp reads were truncated at any site receiving an average quality score <20 over a 10 bp sliding window, discarding the truncated reads that were shorter than 50bp. (ii) exact barcode matching, 2 nucleotide mismatch in primer matching, reads containing ambiguous characters were removed. (iii) only sequences that overlap longer than 10 bp were assembled according to their overlap sequence. Reads which could not be assembled were discarded. Operational taxonomic units (OTUs) were clustered with 97% similarity cutoff using UPARSE (version 7.1 http://drive5.com/uparse/) and chimeric sequences were identified and removed using UCHIME. The phylogenetic affiliation of each 16S rRNA gene sequence was analyzed by RDP Classifier (http://rdp.cme.msu.edu/) against the silva (SSU123) 16S rRNA database using confidence threshold of 70% (Amato et al., 2013). The raw data has been deposited in the NCBI Sequence Read Archive (SRA) database (BioProject ID: PRJNA494772).

### Statistical Analysis

The data was expressed as mean ± standard error (SE). Statistical differences were analyzed with the parametric or non-parametric unpaired Student’s t-test. SPSS version 19.0 (IBM, Armonk, NY) was used for statistical analysis and p value < 0.05 was considered statistically significant. Analyses were performed on the normalized data set. Statistical analysis of the gut microbiota was performed using the R 3.2.4. Principal Coordinate Analyses (PCoA) were performed by package ape version 3.4 of R version 3.2.4. Alpha diversity (Shannon-Wiener index) was calculated using package Vegan version 2.3-0 of R version 3.2.4. The samples were rarefied for alpha diversity analysis. The beta-diversity was estimated at the OUT level. Kruskal-Wallis rank sum tests and Wilcoxon rank sum tests were performed by package stats version 3.2.4 R version 3.2.4. LDA Effect Size (LEfSE) analyses and cladogram were performed according to Segata et al. (Segata et al., 2011). The differential abundance analyses with LEfSE were estimated at the genus and above level. The GraPhlAn (Graphical Phylogenetic Analysis) visualization of the OTUs were performed according to Asnicar et al. (Asnicar et al., 2015). OTUs were pooled at the genus level. The heatmaps showed differentially abundant taxa.

## Results

### DJB Attenuates HFD-Induced Hepatic Steatosis

**Figure 1:**
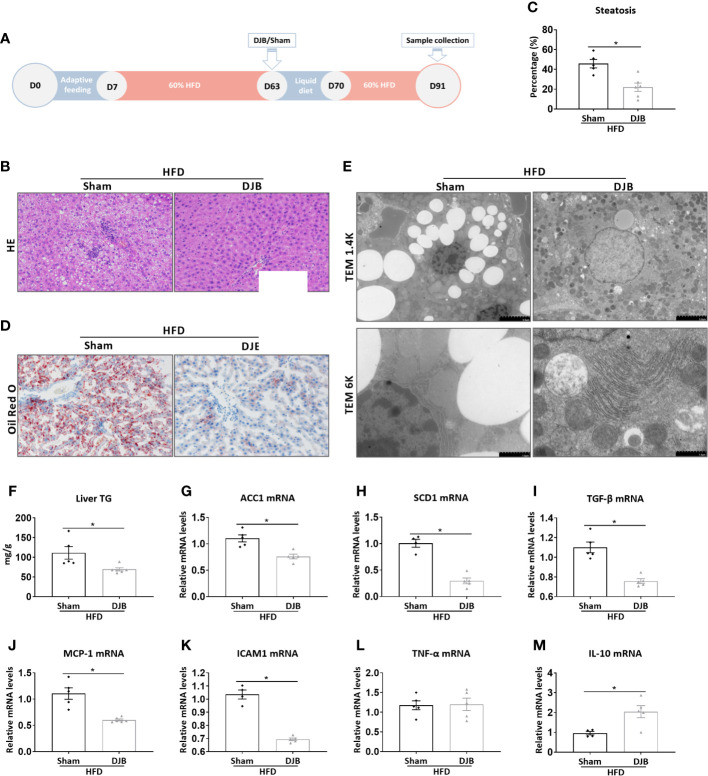
Effects of DJB on hepatic steatosis in HFD-fed rats. **(A)** Experimental design: Male SD rats were fed with a 60% HFD to induce liver injury. At 8 weeks after HFD feeding, the rats were subjected to either DJB or sham operation. HFD was resumed 1 week after the surgery for 3 more weeks. The effect of DJB on hepatic steatosis was evaluated at 4 weeks after surgery. **(B)** Representative photomicrographs of liver histology from an HFD-fed DJB-operated rat (HFD-DJB) and an HFD-fed sham-operated rat (HFD-Sham). Original magnification, x200. **(C)** the percentage of steatotic hepatocytes in HFD-DJB and HFD-Sham rats. Original magnification, x200. **(D)** Representative photomicrographs of Oil Red O staining from a HFD-DJB rat and a HFD-Sham rat. Original magnification, x200. **(E)** Representative images of electron microscopy from a HFD-DJB rat and a HFD-Sham rat. **(F)** Liver triglyceride content in HFD-DJB and HFD-Sham rats. Hepatic mRNA expression of ACC1 **(G)**, SCD1 **(H)**, TGF‐β **(I)**, MCP-1 **(J)**, ICAM1 **(K)**, TNF-α **(L)**, and IL-10 **(M)** in HFD-DJB and HFD-Sham rats. Results are expressed as mean ± SE (HFD-Sham, n=5; HFD-DJB, n=6) and compared by Student’s t-test; *P<0.05 versus the HFD-Sham group. DJB, Duodenal-jejunal bypass surgery; HFD, High fat diet; NAS, NAFLD activity score; TG, triglycerides; ACC1, Acetyl-CoA carboxylase-1; SCD1, Stearoyl-CoA desaturase-1; TGF‐β, Transforming growth factor beta; MCP-1, Monocyte chemotactic protein 1; ICAM1, Intercellular adhesion molecule 1; TNF-α, Tumor necrosis factor alpha; IL-10, Interleukin-10.

To investigate the effect of DJB on hepatic steatosis, male SD rats were fed with a 60% HFD. At 8 weeks after HFD feeding, the rats were subjected to either DJB or sham operation. HFD was resumed 1 week after the surgery for 3 more weeks. The effect of DJB on hepatic steatosis was evaluated at 4 weeks after surgery (**Figure 1A**). As shown in **Figure 1B**, the liver of HFD-fed rats had an extensive accumulation of fat droplets, hepatocyte ballooning and lobular inflammation. DJB reduced histological NAFLD in the liver, which was accompanied by a significant decrease in the percentage of steatotic hepatocytes (**Figure 1C**, P<0.05). Similarly, Oil Red O staining (**Figure 1D**) and electron microscopy (**Figure 1E**) also revealed a significantly improvement in hepatic steatosis after DJB. Consistently, DJB also led to a 34.2% decrease in hepatic triglyceride content (**Figure 1F**). ACC1 (Acetyl-CoA carboxylase 1) and SCD1 (Stearoyl-CoA desaturase-1) are two important enzymes in fatty acid metabolism (Paton and Ntambi, 2009; Goedeke et al., 2018). As shown in **Figures 1G, H**, DJB significantly reduced hepatic mRNA expression of ACC1 and SCD1 in HFD-fed rats. Hepatic mRNA expression of TGF‐β (Transforming growth factor beta), a critical mediator in fibrogenesis, was also decreased after DJB (**Figure 1I**). MCP-1 (Monocyte chemotactic protein 1) is an important chemokine that regulates migration and infiltration of macrophages. DJB significantly downregulated hepatic MCP-1 mRNA expression in HFD-fed rats (**Figure 1J**). Similarly, DJB also reduced ICAM1 (Intercellular adhesion molecule 1) gene expression in the liver of HFD-fed rats (**Figure 1K**). Hepatic mRNA expression of pro-inflammatory cytokine TNF-α (Tumor necrosis factor alpha) did not significantly change (**Figure 1L**), while anti-inflammatory cytokine IL-10 (Interleukin-10) increased markedly after DJB (**Figure 1M**).

### HFD Feeding Induces Significant Changes in the Intestinal Microbiota

**Figure 2:**
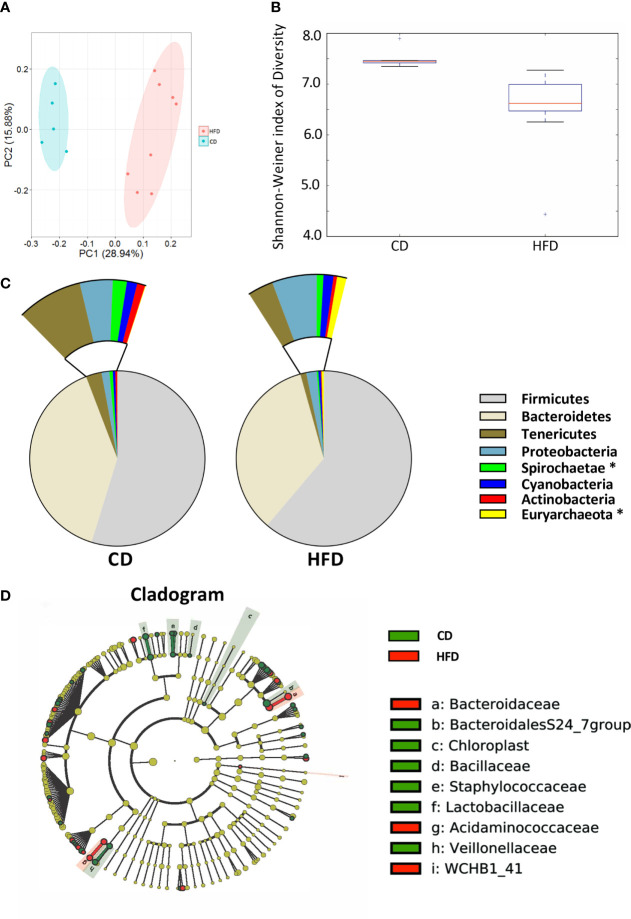
Bacterial 16S rRNA-based analysis of the intestinal microbiota of CD- and HFD-fed rats. **(A)** PCoA plot showing the unweighted UniFrac distance between CD- and HFD-fed rats. Each rat is identified by a point. **(B)** Shannon-Weiner index representing the alpha-diversity in CD- and HFD-fed rats. **(C)** Pie charts showing the relative abundance of phyla in the fecal microbiota of CD and HFD-fed rats (Kruskall Wallis test). **(D)** Cladogram showing the taxa most differentially associated with CD- (green) or HFD-fed (red) rats (Wilcoxon rank-sum test). Circle sizes in the cladogram plot are proportional to bacterial abundance. The circles represent, going from the inner circle to the outer circle: phyla, class, order, family and genus. CD, n=6; HFD, n=8 CD, Control diet; HFD, High fat diet; OTU, operational taxonomic unit.

After 12 weeks control diet (CD, n=5) or 60% high fat diet (HFD, n=8), the colonic contents of each group were harvested and used to analyze the composition and diversity of intestinal microbiota. We obtained an average of 47,551 sequence reads per sample for the V4–V5 region of the bacterial 16S rRNA gene before quality control. And after quality control, an average of 43,439 sequence reads per sample was rarefied to be bacterial genes, corresponding to a total of 11184 OTUs. Principal coordinate analysis (PCoA) showed the compositional structure of the microbiome clustered differently according to their diets (**Figure 2A**). The alpha-diversity calculated by the Shannon-Weiner index, showed that bacterial species diversity was significantly different between HFD-fed and CD-fed rats (P=0.007, **Figure 2B**). The heatmap analysis indicated that the profile of bacterial genera in CD-fed rats was significantly different from the profile in HFD-fed rats (P<0.05, **Supplementary Figure 2**). HFD appeared to decrease bacterial species diversity. The phyla analysis showed CD-fed rats had a larger proportion of Spirochaetae, whereas HFD-fed rats had a larger proportion of Euryarchaeota (**Figure 2C**). The specific taxa that were altered by HFD feeding are shown in **Figure 2D** and **Supplementary Figure 2**.

### DJB Alters the Intestinal Microbiota in HFD-Fed Rats

**Figure 3:**
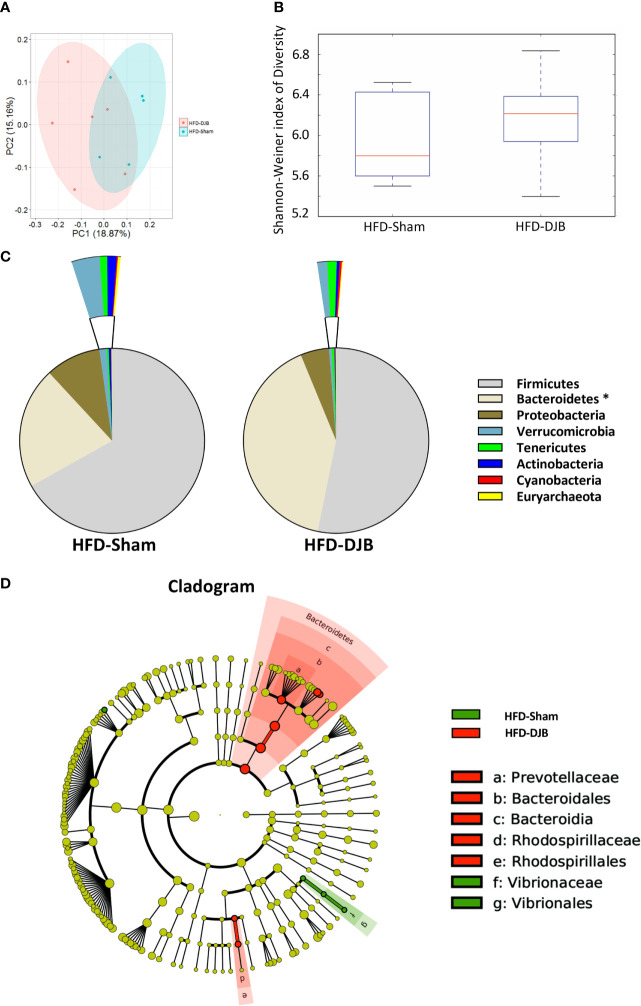
Effects of DJB on the intestinal microbiota in HFD-fed rats. **(A)** PCoA plot showing the unweighted UniFrac distance between HFD-fed DJB-operated (HFD-DJB) and HFD-fed sham-operated (HFD-Sham) rats. Each rat is identified by a point. **(B)** Shannon-Weiner index representing the alpha-diversity in HFD-DJB and HFD-Sham rats. **(C)** Pie charts showing the relative abundance of phyla in the fecal microbiota of HFD-DJB and HFD-Sham rats (Kruskall Wallis test). **(D)** Cladogram showing the taxa most differentially associated with HFD-Sham (green) or HFD-DJB (red) rats (Wilcoxon rank-sum test). Circle sizes in the cladogram plot are proportional to bacterial abundance. The circles represent, going from the inner circle to the outer circle: phyla, class, order, family and genus. HFD-Sham, n=5; HFD-DJB, n=6. Duodenal-jejunal bypass surgery; HFD, High fat diet; OTU, operational taxonomic unit.

Four weeks after DJB (HFD-DJB, n=6) or SHAM (HFD-SHAM, n=5), the colonic contents of each group were harvested and used to analyze the composition and diversity of intestinal microbiota. We obtained an average of 52,744 sequence reads per sample for the V4–V5 region of the bacterial 16S rRNA gene before quality control. And after quality control, an average of 47,062 sequence reads per sample was rarefied to be bacterial genes, corresponding to a total of 6648 OTUs. The intestinal microbiota of DJB- and sham-operated rats clustered differently (**Figure 3A**). DJB did not increase bacterial species diversity in HFD-fed rats (P=0.527, **Figure 3B**). The heatmap analysis indicated that the profile of bacterial genera in DJB-operated rats was significantly different than the profile in sham-operated rats (P<0.05, **Supplementary Figure 4**). The phyla analysis showed DJB increased the proportion of Bacteroidetes in HFD-fed rats (**Figure 3C**). The specific taxa that were altered by DJB are shown in **Figure 3D** and **Supplementary Figure 5**.

### Transplantation of the Fecal Content From DJB Rats Attenuates HFD-Induced Hepatic Steatosis

**Figure 4:**
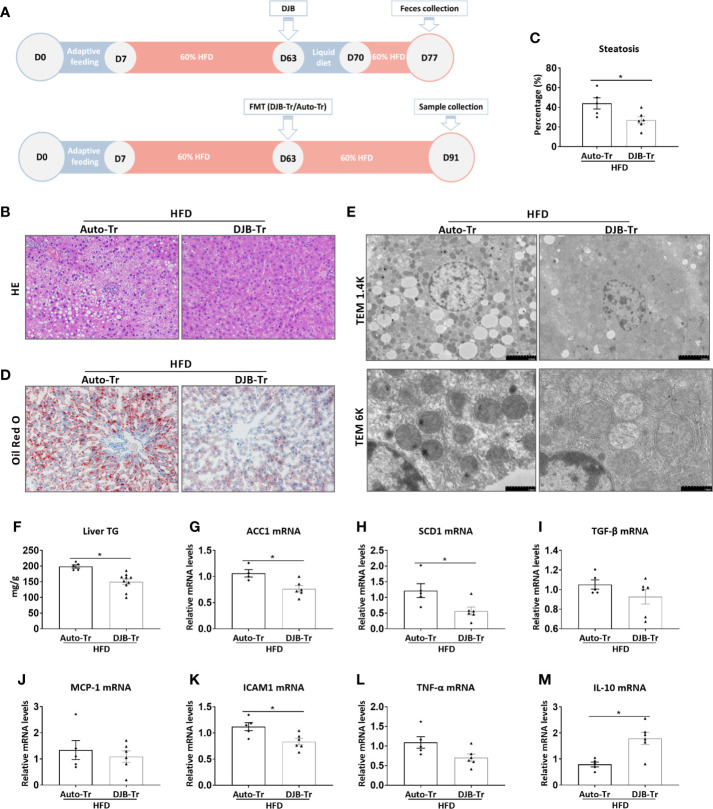
Effects of FMT on hepatic steatosis in HFD-fed rats. **(A)** Feces were collected from HFD-DJB rats at 2 weeks after surgery. These feces were then transplanted to HFD-fed rats without DJB at 8 weeks after HFD feeding (HFD-DJB-Tr). The control rats received transplantation of their own feces (HFD-Auto-Tr). After fecal transplantation, the animals were maintained on the HFD for 4 more weeks. **(B)** Representative photomicrographs of liver histology from an HFD-fed rat receiving DJB FMT (HFD-DJB-Tr) and an HFD-fed rat receiving auto FMT (HFD-Auto-Tr). Original magnification, x200. **(C)** the percentage of steatotic hepatocytes in HFD-DJB-Tr and HFD-Auto-Tr rats. Original magnification, x200. **(D)** Representative photomicrographs of Oil Red O staining from aHFD-DJB-Tr rat and a HFD-Auto-Tr rat. Original magnification, x200. **(E)** Representative images of electron microscopy from an HFD-DJB-Tr rat and an HFD-Auto-Tr rat. **(F)** Liver triglyceride content in HFD-DJB-Tr and HFD-Auto-Tr rats. Hepatic mRNA expression of ACC1 **(G)**, SCD1 **(H)**, TGF‐β **(I)**, MCP-1 **(J)**, ICAM1 **(K)**, TNF-α **(L)**, and IL-10 **(M)** in HFD-DJB-Tr and HFD-Auto-Tr rats. Results are expressed as mean ± SE (HFD-Auto-Tr, n=5; HFD-DJB-Tr, n=10) and compared by Student’s t-test; *p < 0.05 versus the HFD-Auto-Tr group. DJB, Duodenal-jejunal bypass surgery; HFD, High fat diet; NAS, NAFLD activity score; ACC1, Acetyl-CoA carboxylase-1; SCD1, Stearoyl-CoA desaturase-1; TGF‐β, Transforming growth factor beta; MCP-1, Monocyte chemotactic protein 1; ICAM1, Intercellular adhesion molecule 1; TNF-α, Tumor necrosis factor alpha; IL-10, Interleukin-10.

To investigate the role of the gut microbiota in DJB-associated improvement of hepatic steatosis in HFD-fed rats, feces were collected from HFD-DJB rats at 2 weeks after surgery. These feces were then transplanted to HFD-fed rats without DJB at 8 weeks after HFD feeding (HFD-DJB-Tr). The control rats received transplantation of their own feces (HFD-Auto-Tr). After fecal transplantation, the animals were maintained on the HFD for 4 more weeks (**Figure 4A**). The analysis of liver lesions indicated that transplantation of the fecal content from DJB rats was sufficient to alleviate hepatic steatosis in HFD-fed rats as shown by HE staining (**Figure 4B**), the percentage of steatotic hepatocytes (**Figure 4C**), Oil Red O staining (**Figure 4D**), electron microscopy (**Figure 4E**), and hepatic triglyceride content (**Figure 4F**). Hepatic mRNA expression levels of ACC1 and SCD1 were also significantly lower in the HFD-DJB-Tr group than the HFD-Auto-Tr group (**Figures 4G, H**). In terms of inflammatory markers, rats received fecal transplants from DJB rats had lower ICAM1 (**Figure 4K**) and higher IL-10 (**Figure 4M**) mRNA expression in the liver as compared with those received fecal autotransplants. However, there were no statistically significant differences in TGF‐β (**Figure 4I**), MCP-1 (**Figure 4J**) and TNF-α (**Figure 4L**) mRNA expression between HFD-DJB-Tr and HFD-Auto-Tr rats.

### Microbiota Associated With Improvement of Hepatic Steatosis in DJB-Operated Rats

**Figure 5:**
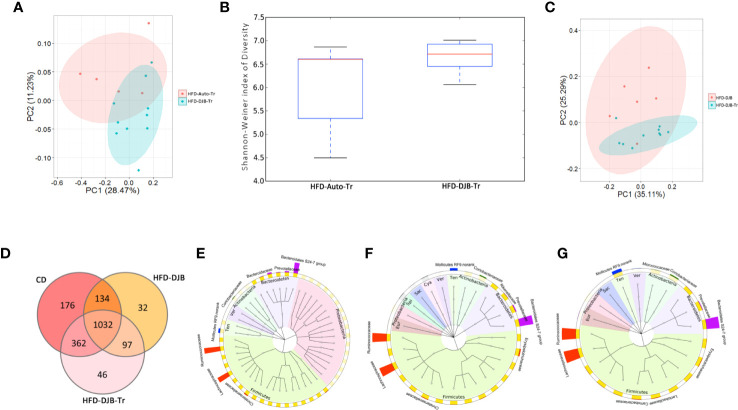
Microbiota associated with improvement of hepatic steatosis in DJB-operated rats. **(A)** PCoA plot showing the unweighted UniFrac distance between HFD-DJB-Tr and HFD-Auto-Tr rats. Each rat is identified by a point. **(B)** Shannon-Weiner index representing the alpha-diversity in HFD-DJB-Tr and HFD-Auto-Tr rats. **(C)** PCoA plot showing the weighted UniFrac distance between HFD-DJB-Tr and HFD-DJB rats. Each rat is identified by a point. **(D)** Venn diagram based on OTU distribution between CD-fed, HFD-DJB and HFD-DJB-Tr rats. **(E)** The GraPhlAn (Graphical Phylogenetic Analysis) visualization of the OTUs that were not significantly different among CD-fed, DJB and DJB-Tr rats (P>0.05, Kruskall Wallis test). **(F)** TheGraPhlAn visualization of the OTUs that were significantly decreased in HFD-fed rats as compared with CD-fed rats (P<0.05, Wilcoxn rank sum test). **(G)** The GraPhlAn visualization of the overlapping OTUs between **(E, F)** The GraPhlAn visualizations were constructed based on the phylogenetic tree of the microbes in the samples. The ring around the phylogenetic tree is the heatmap of the microbial community. The heatmap is based on the phylum level. The intensity of the color in the heatmap represents the relative abundance of the identified phyla. The outer ring around the heatmap is the barplot of the microbial community based on the family level. HFD-Auto-Tr, n=5; HFD-DJB-Tr, n=10. DJB, Duodenal-jejunal bypass surgery; HFD, High fat diet; OTU, operational taxonomic unit; Cya, cyanobacteria; Eur, Euryarchaeota; Sac, saccharibacteria; Spi, spirochaetae; Ten, Tenericutes; Ver, verrucomicrobia.

After 4 weeks fecal transplantation, the colonic contents of HFD-DJB-Tr (n=10) or HFD-Auto-Tr (n=5) group were harvested and used to analyze the composition and diversity of intestinal microbiota. We obtained an average of 47,575 sequence reads per sample for the V4–V5 region of the bacterial 16S rRNA gene before quality control. And after quality control, an average 42,049 sequence reads per sample was rarefied to be bacterial genes, corresponding to a total of 11,698 OTUs. As shown in **Figure 5A**, the intestinal microbiota of HFD-DJB-Tr and HFD-Auto-Tr rats clustered differently. The pairwise one-way analysis of similarities (ANOSIM) test indicated that the intestinal microbiota was significantly different between HFD-DJB-Tr and HFD-Auto-Tr rats (R=0.3585, P=0.01, based on the unweighted unifrac distance). Compared with HFD-Auto-Tr rats, HFD-DJB-Tr rats appeared to have higher bacterial species diversity (P=0.054, **Figure 5B**). The heatmap analysis indicated that the profile of bacterial genera in HFD-DJB-Tr rats was significantly different than the profile in HFD-Auto-Tr rats (P<0.05, **Supplementary Figure 6**). **Supplementary Figures 7A, B** show the special taxa that were significantly different between HFD-DJB-Tr and HFD-Auto-Tr rats. The intestinal microbiota of the HFD-DJB-Tr rats still different from that of the DJB rats (**Figure 5C**). The Venn diagram of the sequenced intestinal microbiota illustrates the distribution of operational taxonomic unit (OTUs) shared by CD-fed, HFD-DJB and HFD-DJB-Tr rats: a total of 1,032 OTUs were the same (**Figure 5D**). To pinpoint the specific taxa responsible for the beneficial effects of DJB and DJB-Tr on hepatic steatosis in HFD-fed rats, we first identified the relative abundance of OTUs that were not significantly different among CD-fed, DJB and DJB-Tr rats (**Figure 5E**, Kruskal-Wallis tests, P>0.05) and then isolated the relative abundance of OTUs that were significantly decreased in HFD-fed rats as compared with CD-fed rats (**Figure 5F**, Wilcoxon tests, P<0.05). The overlapping OTUs were shown in **Figure 5G**. These bacteria were mostly of the Firmicutes and Bacteroidetes phylum. The specific families in the Firmicutes phylum mainly included Lachnospiraceae, Ruminococcaceae, Carnobacteriaceae, Lactobacillaceae, Erysipelotrichaceae, Bacillaceae, PlanococcaceaeamdChristensenellaceae (**Supplementary Figure 8A**). The specific families in the Bacteroidetes phylum included Bacteroidales S24-7 group, Prevotellaceae and Porphyromonadaceae (**Supplementary Figure 8B**).

## Discussion

In the current study, we found that DJB alleviated hepatic steatosis in HFD-fed rats, which was associated with substantial compositional changes in the gut microbiota. More importantly, treatment of rats with HFD-induced liver lesions by fecal transplant from DJB-operated HFD-fed rats also attenuated hepatic steatosis. These findings accentuate the role of the gut microbiota in HFD-induced NASH as well as DJB-associated improvement of hepatic steatosis under such a condition.

Despite rising prevalence, effective treatments for NAFLD remain limited. There is still no approved pharmacological treatment for NAFLD (Augustin et al., 2017). Current treatment strategies for NAFLD focus on weight reduction, controlling diabetes, and lowering levels of cholesterol and triglycerides. Recommendations include eating a healthy diet, exercising regularly, and avoiding alcohol. Gastrointestinal bypass surgery can lead to significant improvements in NAFLD. A recent systematic review and meta-analysis of 29 relevant studies showed that bariatric surgery resulted in resolution or significant improvement of steatosis, nonalcoholic steatohepatitis, and fibrosis in a majority of patients (Bower et al., 2015). The mechanism underlying these beneficial effects is complex and not fully understood. Weight loss, increased insulin sensitivity, alterations in gut hormone production and decreases in dyslipidemia and inflammation have all been proposed to contribute (Hafeez and Ahmed, 2013). In this study, we found that fecal transplant from DJB-operated rats can effectively attenuate HFD-induced NASH, indicating alterations in the gut microbiota after DJB are sufficient to improve NASH. As with any major surgery, gastrointestinal bypass surgery can pose potential health risks, both in the short term and long term. And currently, NAFLD *per se* is not an indication for bariatric surgery (Hafeez and Ahmed, 2013). The findings in this study suggest that fecal transplant may uphold the benefits of bariatric surgery without exposing patients to the potential surgery-related risks. However, it may not be easy to get feces from patients with DJB. Therapeutic approaches using specific microbiota appear to be more practical for the treatment of human NALFD.

Recent lines of evidence suggest a close relationship between the gut microbiota and the development of NAFLD (Le Roy et al., 2013; Mehal, 2013; Chassaing et al., 2014; Mardinoglu et al., 2018). The gut microbiota may affect the development and progression of NAFLD, both by influencing risk factors for NAFLD and by direct effects on fat accumulation in the liver. Obesity and type 2 diabetes are important risk factors for NAFLD. Obesity is the result of excess caloric intake compared with expenditure. Gut bacteria can either increase or decrease the amounts of digestible sources of energy, particularly monosaccharides and short-chain fatty acids, thereby regulating the amount of calories absorbed from the gut (Turnbaugh et al., 2006). A study of human twin pairs (mostly monozygotic) has shown that lean mice who were fed feces from the fat human twins became fat, while feces from the lean human twins allowed the mice to remain lean (Ridaura et al., 2013). When the fat and lean mice were housed together, the fat mice’s gut flora came to resemble the flora of the lean mice and they became lean. The gut microbiota can also regulate insulin sensitivity. A gut microbiome that produces relatively more acetate and less butyrate increases insulin resistance (Devaraj et al., 2013; Perry et al., 2016). A human study showed that small intestinal infusions of feces from lean male donors to treatment-naïve individuals with metabolic syndrome increased the insulin sensitivity of the recipients, along with levels of butyrate-producing microbiota (Vrieze et al., 2012). The gut microbiome can also induce the gut epithelial barrier dysfunction, which, in turn, leads to microbial translocation and subsequent activation of the innate immune system (Van Olden et al., 2015; Hartmann et al., 2019). The resulting systemic inflammation increases insulin resistance.

Although humans and rodents have significantly different microbial strains and species in their gastrointestinal tracts, many aspects of the microbial response to gastrointestinal bypass surgery are conserved among humans, rats, and mice (Ley et al., 2005; Ley et al., 2008). An excellent study by Liou et al. showed a substantial increase in the amount of verrucomicrobia (Akkermansia) and gammaproteobacteria (Escherichia) was observed in fecal samples from RYGB-treated mice, which is similar to the microbial changes found in human patients after gastric bypass surgery (Liou et al., 2013). Although the gut microbiome is suspected to play a role in the development of NALFD, clinical studies examining the alteration of the gut microbiome in patients with NAFLD have yielded significant heterogeneity (Boursier and Diehl, 2016; Roychowdhury et al., 2018). Nonetheless, a significant association between the presence of NASH and lower percentage Bacteroidetes has been shown in a recent human study (Mouzaki et al., 2013). Moreover, decreased Firmicutes numbers were found in obese children and adolescents with NASH (Zhu et al., 2013).

HFD can lead to dynamic, qualitative and quantitative changes in the gut microbiome (dysbiosis). The altered gut microbiome may be a major pathogenic driver of hepatic inflammation and associated liver diseases. Maintaining microbiota diversity in the gut is vital for overall health (Cheng et al., 2017). In this study, we also found that HFD feeding resulted in significant loss of overall microbial diversity. More importantly, we demonstrate that the altered microbiota after DJB surgery is sufficient to trigger an improvement in NAFLD. Our results corroborate a recent study showing changes in the gut microbiota are partially responsible for the weight loss and reduced adiposity observed in obese mice following RYGB surgery (Liou et al., 2013). Mice that underwent RYGB surgery had significant weight loss and a characteristic change in the gut microbiome, whereas mice that underwent the sham surgery did not. Transfer of bacteria from mice that underwent RYGB surgery to mice that underwent the sham surgery resulted in weight loss, although not as great as seen following RYGB surgery. However, dissecting the specific role of the gut microbiome in this process remains a great challenge. The host genes, gut microbial genes, and diet share a complex set of interdependencies. We are still at a very early stage of understanding the complicated crosstalk among them. And it is unlikely that a single species of gut bacteria plays a dominant role in the development and progression of NASH. Nonetheless, further isolation of specific strains from the fecal microbiota will be essential to validate their role in protecting against HFD-induced liver lesions.

This study has several limitations. First of all, the beneficial effect of fecal transplantation was examined at 4 weeks after transplantation but no further; therefore, it is not clear how long the beneficial effect of fecal transplantation would last. Then, the changes in the intestinal microbiota was only assessed at one time point without the longitudinal analysis. Moreover, the transplantation experiment was done only with those collected from the rats that received DJB, not control diet-fed rats. In addition, we only had 5-6 rats per group for some experiments. Considering the individual differences in the results of animal experiments, this number was relatively low. Finally, we found hepatic steatosis was improved at 4 weeks after DJB in HFD-fed rats. We chose the middle point (2 weeks) to collect feces for the fecal microbiota transplantation. However, the optimal time point for feces collection remains to be determined.

In summary, microbial community structure is significantly altered after DJB surgery. Alterations in the gut microbiota after DJB surgery are sufficient to attenuate hepatic steatosis and inflammation in HFD-fed rats. These findings have given us preliminary insights to the potential contribution of the gut microbiota in bariatric surgery-associated improvement in NAFLD. They support the idea that targeting the gut microbiota could be a promising approach for preventing or treating human NAFLD.

## Data Availability Statement 

The datasets generated during this study were deposited at the NCBI’s small read archive (BioProject ID: PRJNA494772).

## Ethics Statement

The animal study was reviewed and approved by Institutional Animal Care and Use Committee of the Ethics Committee of Xi’an Jiaotong University Health Science Center.

## Author Contributions

YG: Acquisition of data, analysis and interpretation of data, and statistical analysis. JZ: Acquisition of data and drafting of the manuscript. XX: Analysis and interpretation of data. YR, XY, JY, and TW: Acquisition and analysis of data. ZW: Critical revision of the manuscript. YL: Significant intellectual input and critical revision of the manuscript. RW: Study concept and design, drafting of the manuscript, and critical revision of the manuscript. All authors contributed to the article and approved the submitted version.

## Funding

This work was supported by grants from the National Nature Science Foundation of China (no. 81770491), the Innovation Capacity Support Plan of Shaanxi Province (no. 2020TD-040), the Ministry of Education Innovation Team Development Program of China (no. IRT16R57) and the Education Department of Hubei Province (no. D20152103).

## Conflict of Interest

The authors declare that the research was conducted in the absence of any commercial or financial relationships that could be construed as a potential conflict of interest.

## Abbreviations

DJB, duodenal-jejunal bypass surgery; NAFLD, nonalcoholic fatty liver disease; HFD, high fat diet; CD, control diet; HCC, hepatocellular carcinoma; NASH, nonalcoholic steatohepatitis; RYGB, Roux-en-Y gastric bypass surgery; PCoA, Principal coordinate analysis; OUTs, taxonomic unit; SE, standard error; HFD-DJB-Tr, HFD-fed rats received fecal transplantation from a HFD-DJB rat; HFD-Auto-Tr, HFD-fed rats received transplantation of their own feces; TPN, total parenteral nutrition; FMT, fecal microbiota transplantation; NAS, NAFLD activity score; TG, triglycerides; qRT-PCR, quantitative reverse transcriptase-polymerase chain reaction; ACC1, Acetyl-CoA carboxylase-1; SCD1, Stearoyl-CoA desaturase-1; TGF‐β, Transforming growth factor beta; MCP-1, Monocyte chemotactic protein 1; ICAM1, Intercellular adhesion molecule 1; TNF-α, Tumor necrosis factor alpha; IL-10, Interleukin-10.
