# Supplementary material for: The Role of Gut Microbiota in Duodenal-Jejunal Bypass Surgery-Induced Improvement of Hepatic Steatosis in HFD-Fed Rats
Source: Front Cell Infect Microbiol. 2021 Apr 2;11:640448. doi: 10.3389/fcimb.2021.640448 (PMC8050338; doi:10.3389/fcimb.2021.640448)
Supplement: Supplementary file 1 [file DataSheet_1.docx]

Supplementary Materials for

**The role of gut microbiota in duodenal-jejunal bypass surgery-induced improvement of hepatic steatosis in HFD-fed rats**

Yi Gao, Jia Zhang, Xiao Xiao, Yifan Ren, Xiaopeng Yan, Jing Yue, Tieyan Wang,

Zheng Wu, Yi Lv*, Rongqian Wu*

*Corresponding author: Rongqian Wu, rwu001@mail.xjtu.edu.cn, or Yi Lv, Luyi169@126.com.

**This file includes:**

Supplementary Figure 1

Supplementary Figure 2

Supplementary Figure 3

Supplementary Figure 4

Supplementary Figure 5

Supplementary Figure 6

Supplementary Figure 7

Supplementary Figure 8

**
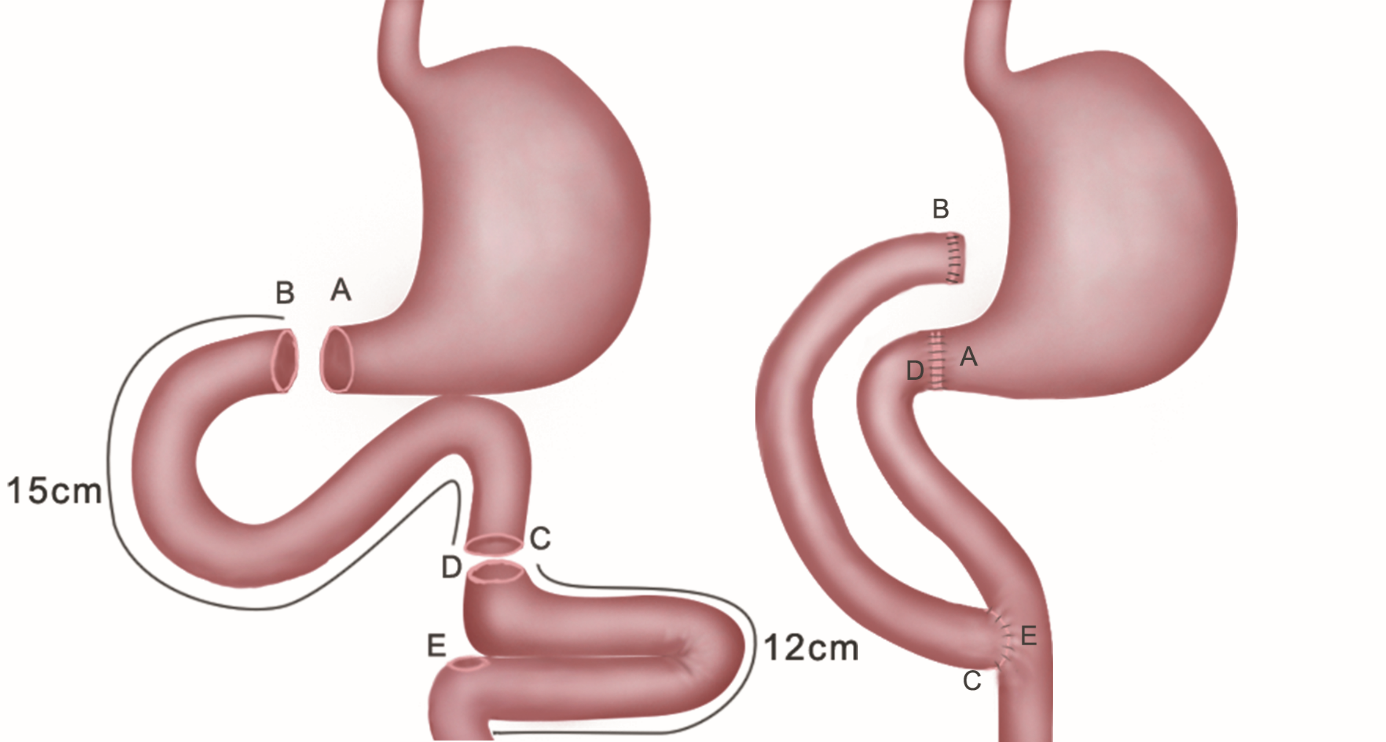
**

**Supplementary Figure 1:** Duodenal-jejunal bypass surgery (DJB) procedure in rats. The duodenum was separated from the stomach (A, B), and bowel continuity was interrupted at the level of the distal jejunum (15 cm from the duodenum. C, D). The distal (D) of the 2 limbs was directly connected to the stomach (gastrojejunal anastomosis, A) and the proximal limb (C) carrying the biliopancreatic juices was reconnected downward to the alimentary limb at a distance of 12 cm from the gastrojejunal anastomosis (E).


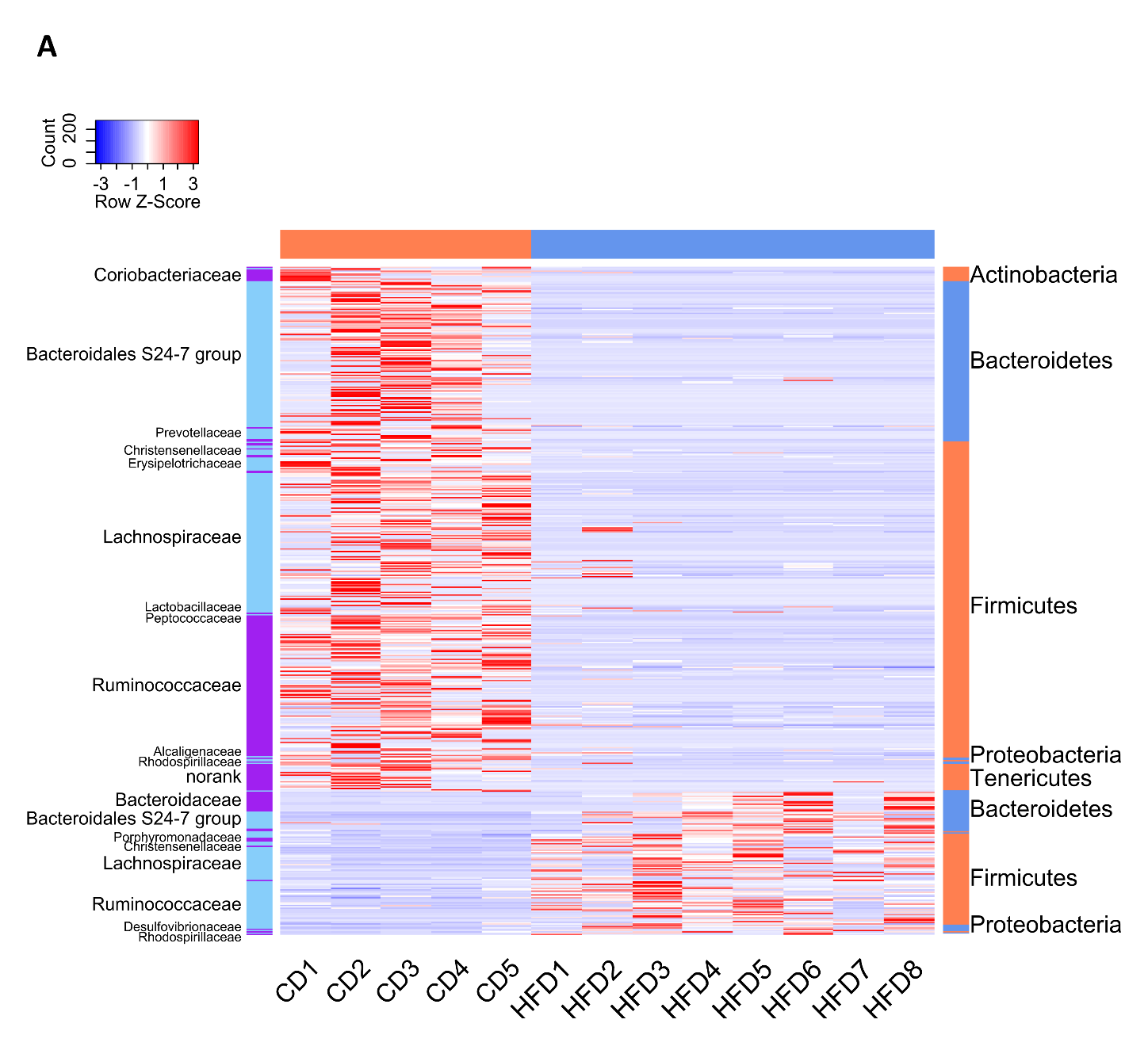


**Supplementary Figure 2:** Male SD rats were fed with either a standard rat chow diet (control diet, CD) or a 60% high-fat diet (HFD) for 12 weeks. The intestinal microbiota was analyzed. Heatmap showing significantly different genera (P < 0.05) of intestinal microbiota community between CD- and HFD-fed rats by Wilcoxon rank-sum test. Side bars depict the phyla and family of the corresponding OTU.


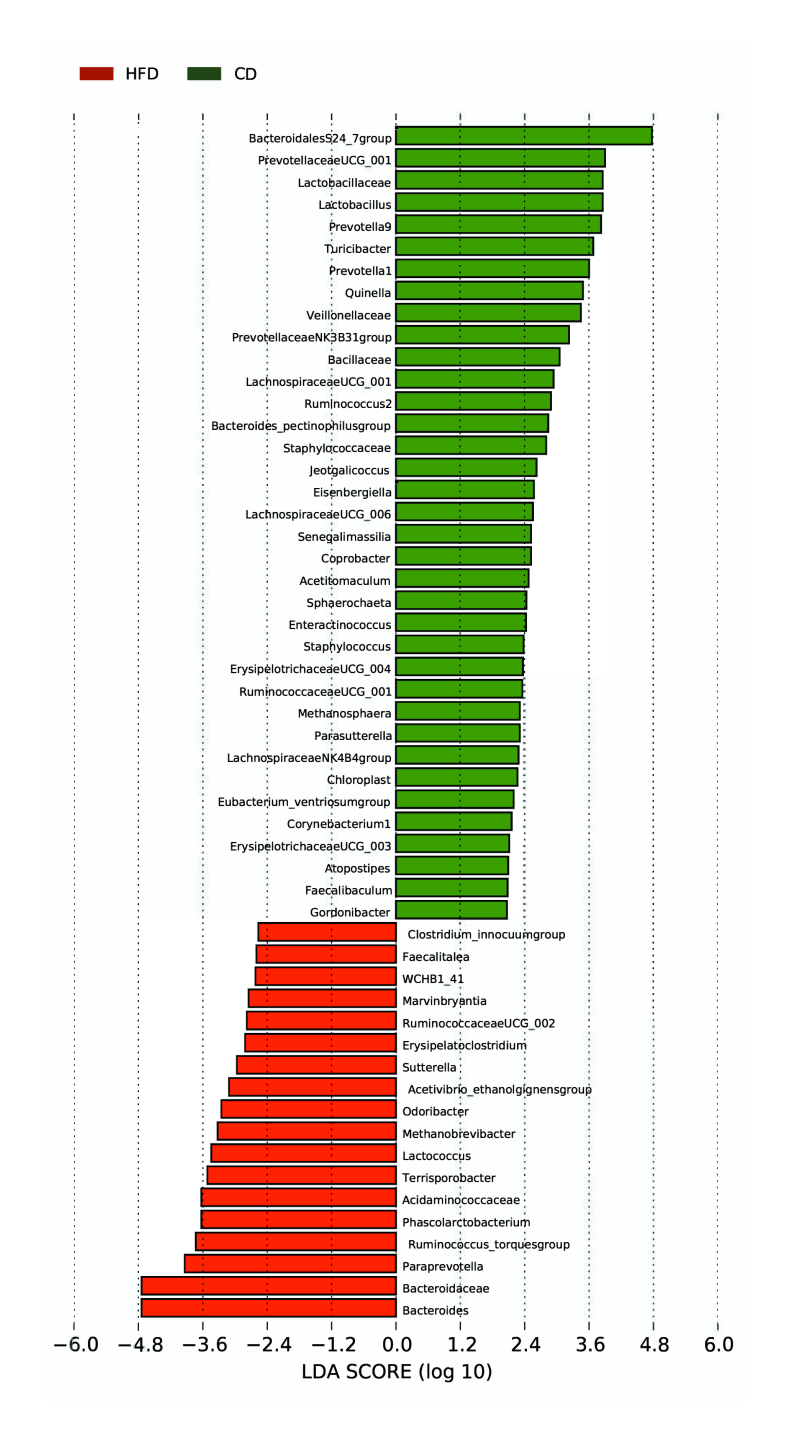


**Supplementary Figure 3:** Male SD rats were fed with either a standard rat chow diet (control diet, CD) or a 60% high-fat diet (HFD) for 12 weeks. The intestinal microbiota was analyzed. Taxa enriched in microbiota from CD- (green) or HFD-fed (red) rats.


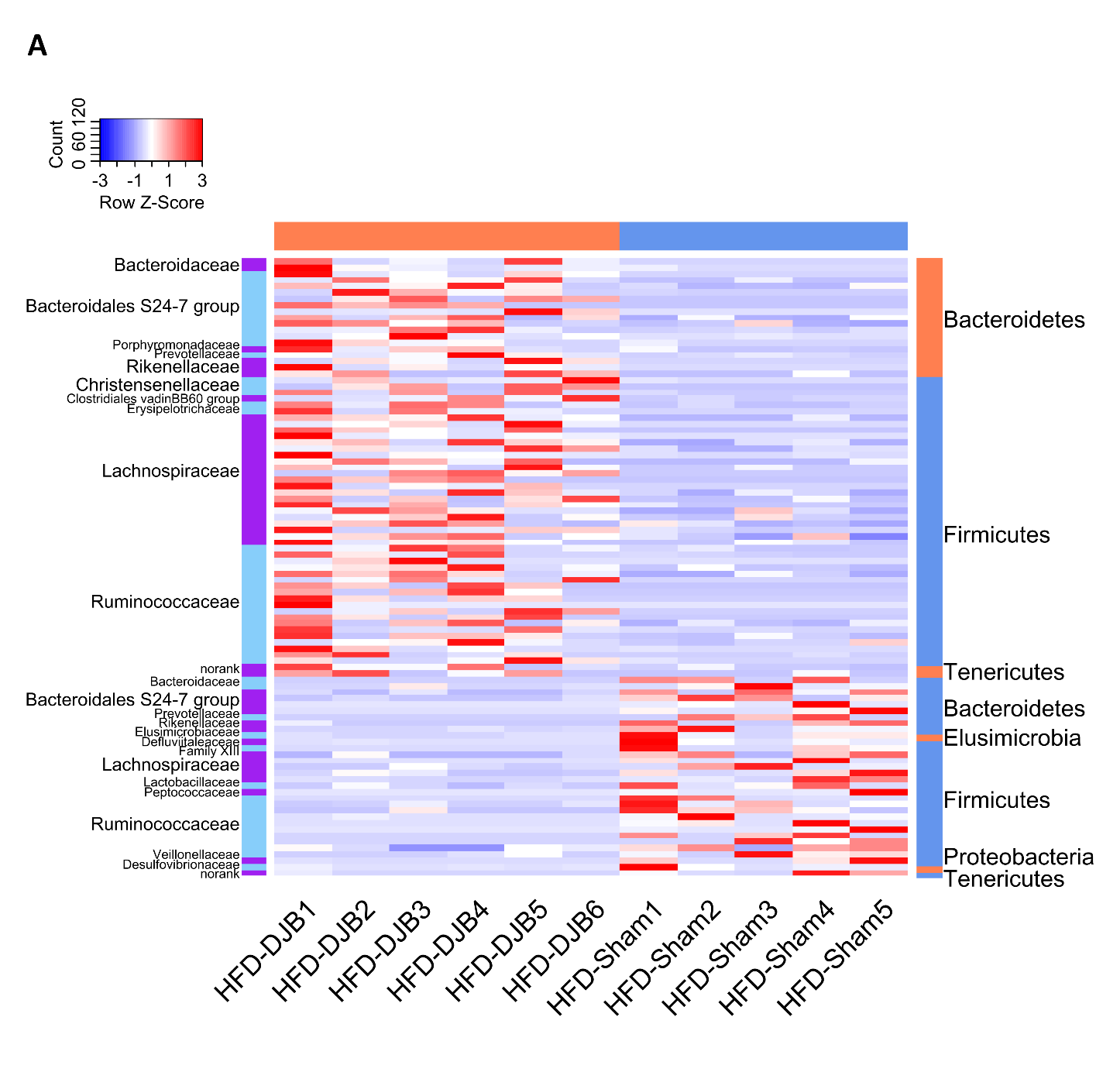


**Supplementary Figure 4:** Male SD rats were fed with a 60% HFD to induce liver injury. At 8 weeks after HFD feeding, the rats were subjected to either DJB or sham operation. HFD was resumed 1 week after the surgery for 3 more weeks. The intestinal microbiota was analyzed at 4 weeks after surgery. Heatmap showing significantly different genera (P < 0.05) of intestinal microbiota community between HFD-DJB and HFD-Sham rats by Wilcoxon rank-sum test. Side bars depict the phyla and family of the corresponding OTU.

**
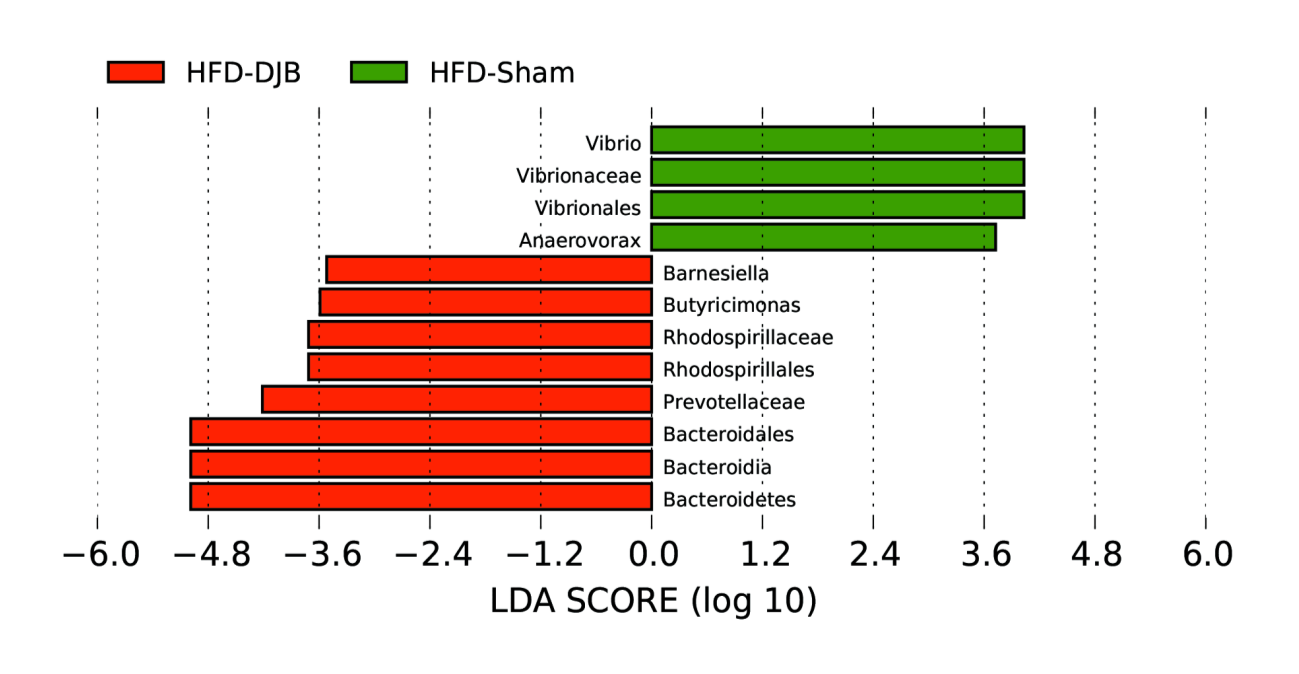
**

**Supplementary Figure 5:** Male SD rats were fed with a 60% HFD to induce liver injury. At 8 weeks after HFD feeding, the rats were subjected to either DJB or sham operation. HFD was resumed 1 week after the surgery for 3 more weeks. The intestinal microbiota was analyzed at 4 weeks after surgery. Taxa enriched in microbiota from HFD-Sham (green) or HFD-DJB (red) rats.


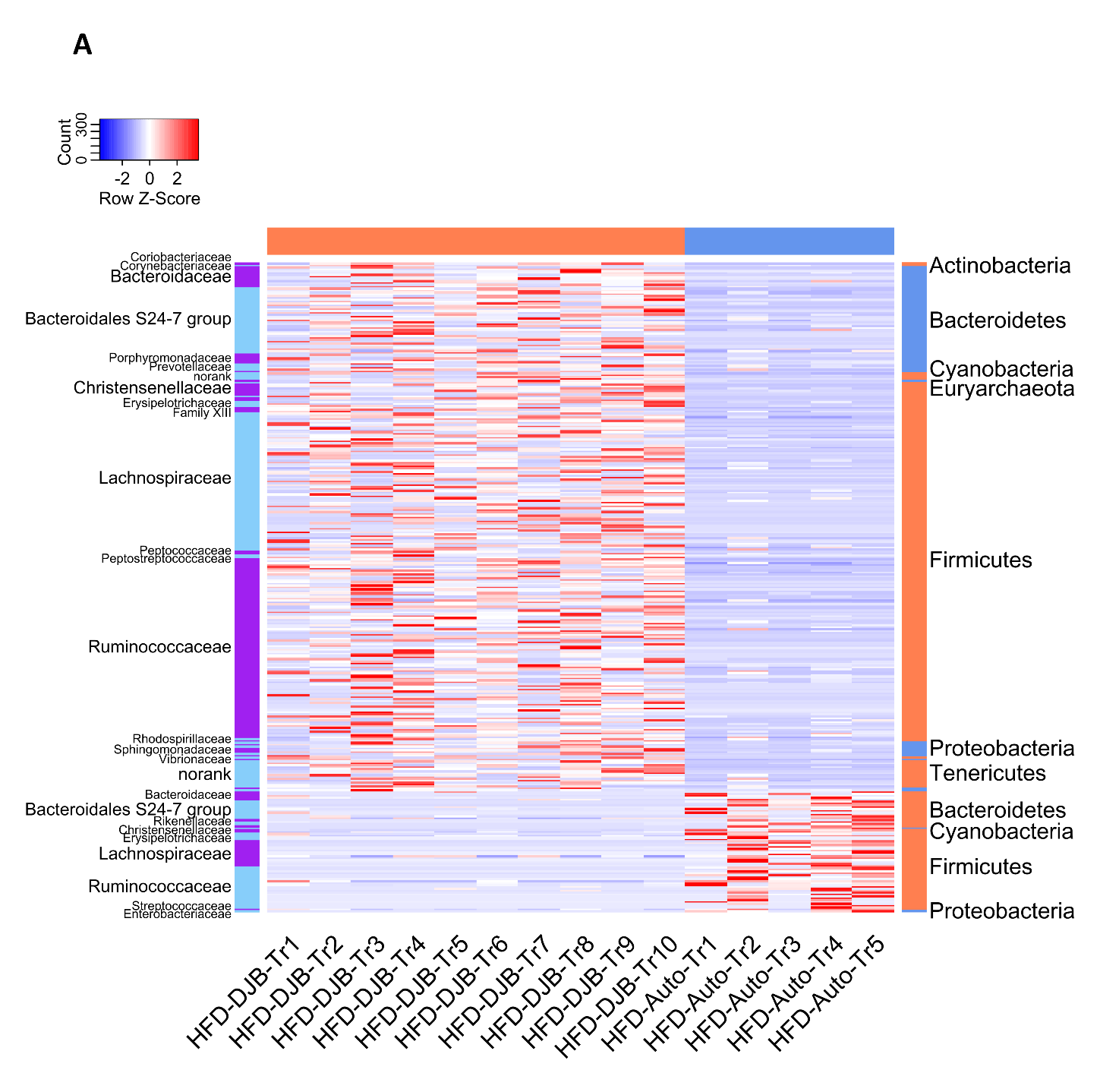


**Supplementary Figure 6:** Feces were collected from HFD-DJB rats at 2 weeks after surgery. These feces were then transplanted to HFD-fed rats without DJB at 8 weeks after HFD feeding (HFD-DJB-Tr). The control rats received transplantation of their own feces (HFD-Auto-Tr). After fecal transplantation, the animals were maintained on the HFD for 4 more weeks. The intestinal microbiota was analyzed. Heatmap showing significantly different genera (P < 0.05) of intestinal microbiota community between HFD-DJB-Tr and HFD-Auto-Tr rats by Wilcoxon rank-sum test. Side bars depict the phyla and family of the corresponding OTU.

**
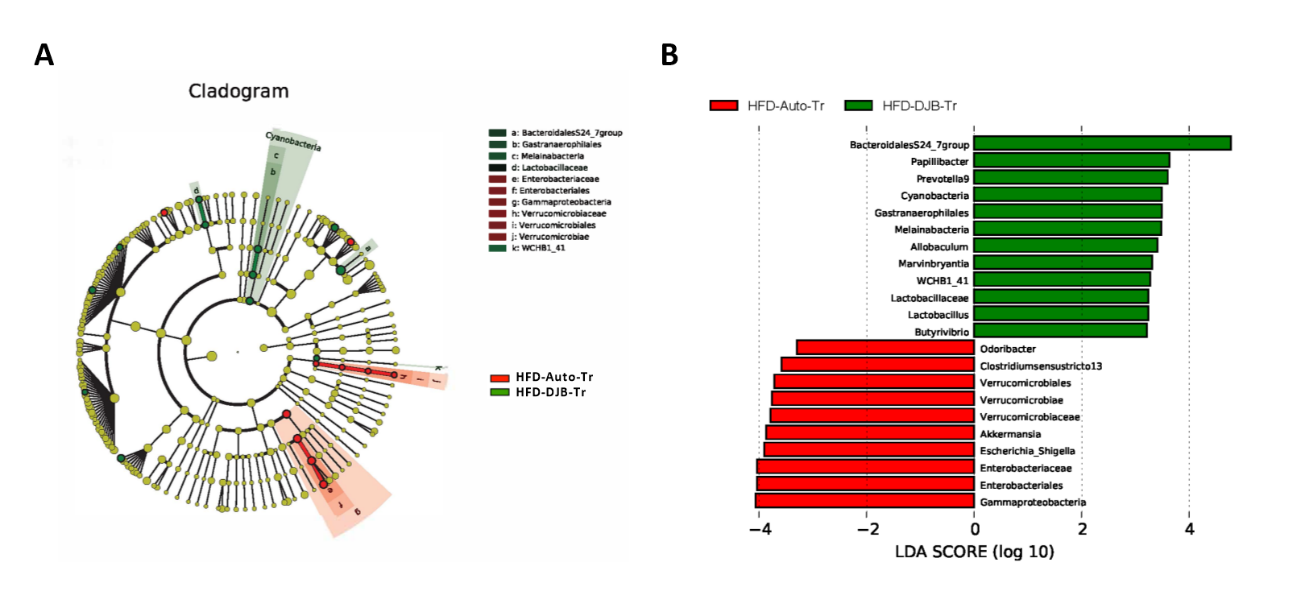
**

**Supplementary Figure 7:** Feces were collected from HFD-DJB rats at 2 weeks after surgery. These feces were then transplanted to HFD-fed rats without DJB at 8 weeks after HFD feeding (HFD-DJB-Tr). The control rats received transplantation of their own feces (HFD-Auto-Tr). After fecal transplantation, the animals were maintained on the HFD for 4 more weeks. The intestinal microbiota was analyzed. Cladogram (**A**) and LDA (**B**) analyses show the specific taxa which were significantly different between HFD-DJB-Tr and HFD-Auto-Tr rats.

**
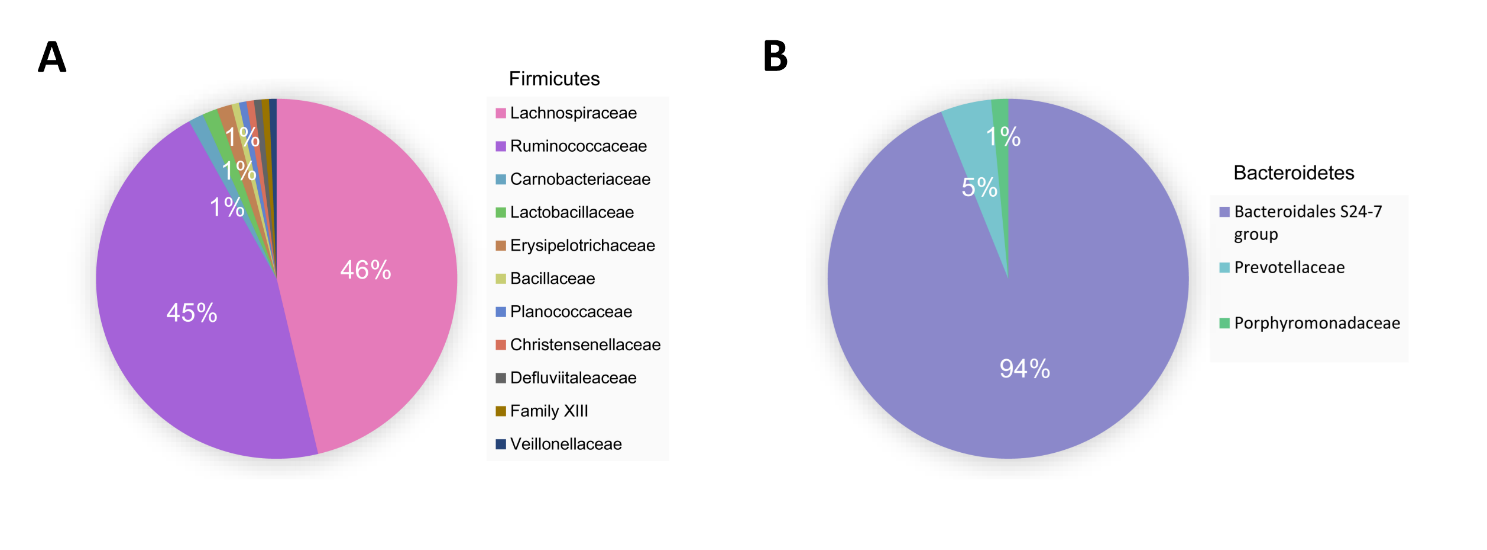
**

**Supplementary Figure 8:** (**A**) The Pie chart of the specific families in the Firmicutes phylum that were not significantly different among CD-fed, DJB and DJB-Tr rats, while significantly decreased in HFD-fed rats as compared with CD-fed rats. The percentages represent the relative abundance of the identified OTUs. (**B**) The Pie chart of the specific families in the Bacteroidetes phylum that were not significantly different among CD-fed, DJB and DJB-Tr rats, while significantly decreased in HFD-fed rats as compared with CD-fed rats. The percentages represent the relative abundance of the identified OTUs.
